# Author Correction: The structure of a red-shifted photosystem I reveals a red site in the core antenna

**DOI:** 10.1038/s41467-020-19953-w

**Published:** 2020-11-20

**Authors:** Hila Toporik, Anton Khmelnitskiy, Zachary Dobson, Reece Riddle, Dewight Williams, Su Lin, Ryszard Jankowiak, Yuval Mazor

**Affiliations:** 1 School of Molecular Sciences, Arizona State University, Tempe, AZ 85287-1604 USA; 2 Biodesign Center for Applied Structural Discovery, Arizona State University, Tempe, AZ 85287 USA; 3 Department of Chemistry, Kansas State University, Manhattan, KS 66506 USA; 4 John M. Cowley Center for High Resolution Electron Microscopy, Arizona State University, Tempe, AZ 85287 USA; 5 Center for Innovations in Medicine at the Biodesign Institute, Arizona State University, Tempe, AZ 85287 USA; 6 Department of Physics, Kansas State University, Manhattan, KS 66506 USA

**Keywords:** Cryoelectron microscopy, Kinetics, Photosystem I

Correction to: *Nature Communications* 10.1038/s41467-020-18884-w, published online 19 October 2020.

The original version of this Article omitted a reference to previous work in “Li, M., Semchonok, D. A., Boekema, E. J. & Bruce, B. D. Characterization and evolution of tetrameric photosystem I from the thermophilic cyanobacterium *Chroococcidiopsis* sp TS-821. *Plant Cell* 26, 1230–1245 (2014)”. This has been added as reference 51 which has been added at the end of the fourth sentence of the third paragraph of the Discussion: “The short loop present … *Chroococcidiopsis* sp. TS-821 (PDBID: 6QWJ)^51^”.

The work in reference 50 was cited in the wrong location. It is now cited at the end of the previous sentence as reference 49: “The red loop we added to the *Synechocystis* WT PSI based on *T. elongatus* structure can also be found in *Thermosynechococcus vulcanus*^46^, *Fischerella thermalis*^47^ and *Nostoc* sp. PCC 7120^48,49^”.

This has been corrected in the PDF and HTML versions of the Article.

