# Supplementary material for: The structure of a red-shifted photosystem I reveals a red site in the core antenna
Source: Nat Commun. 2020 Oct 19;11:5279. doi: 10.1038/s41467-020-18884-w (PMC7573975; doi:10.1038/s41467-020-18884-w)
Supplement: Supplementary file 1 — Supplementary Information [file 41467_2020_18884_MOESM1_ESM.pdf]

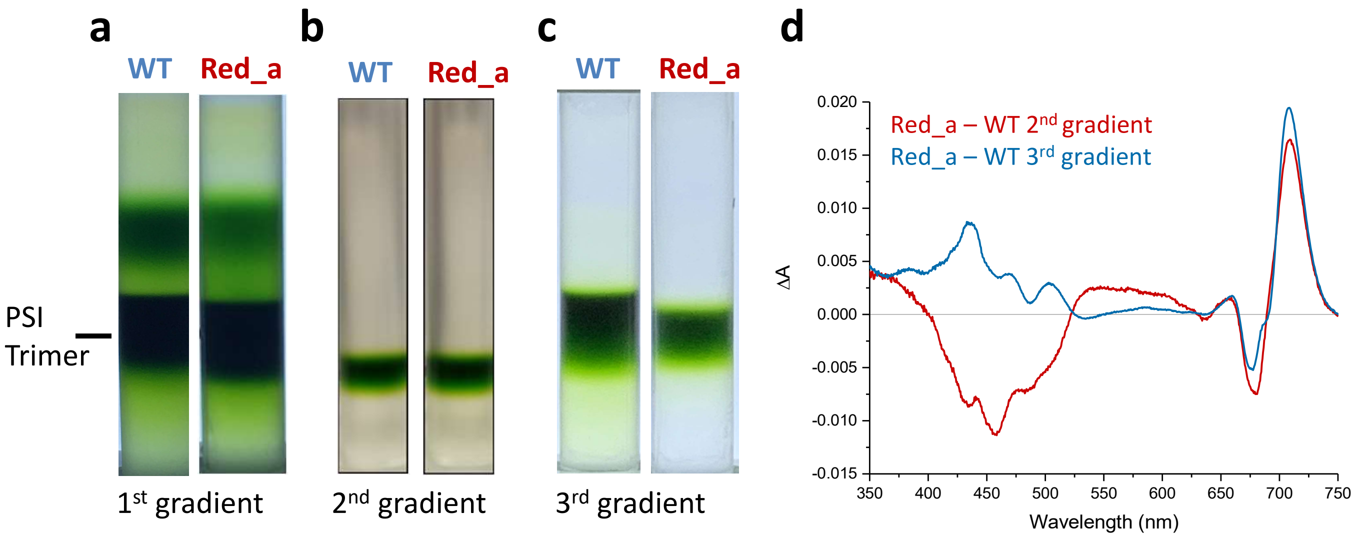

Supplementary figure 1. Steps in sample preparation a. A 10% to 30% sucrose gradient of the main chlorophyll containing fraction from the anion exchange column. **b.** A 2nd sucrose gradient after the PSI trimer band is collected from the 1st gradient, precipitated and loaded on a second sucrose gradient. **c**. A 3rd sucrose gradient purification performed on the main trimer band from the second gradient. **d**. Difference absorbance spectra between the Red_a PSI trimer and the WT PSI trimer from the 2nd gradient in red and the 3rd gradient in blue showing the variability in carotenoids content at the different purification steps.

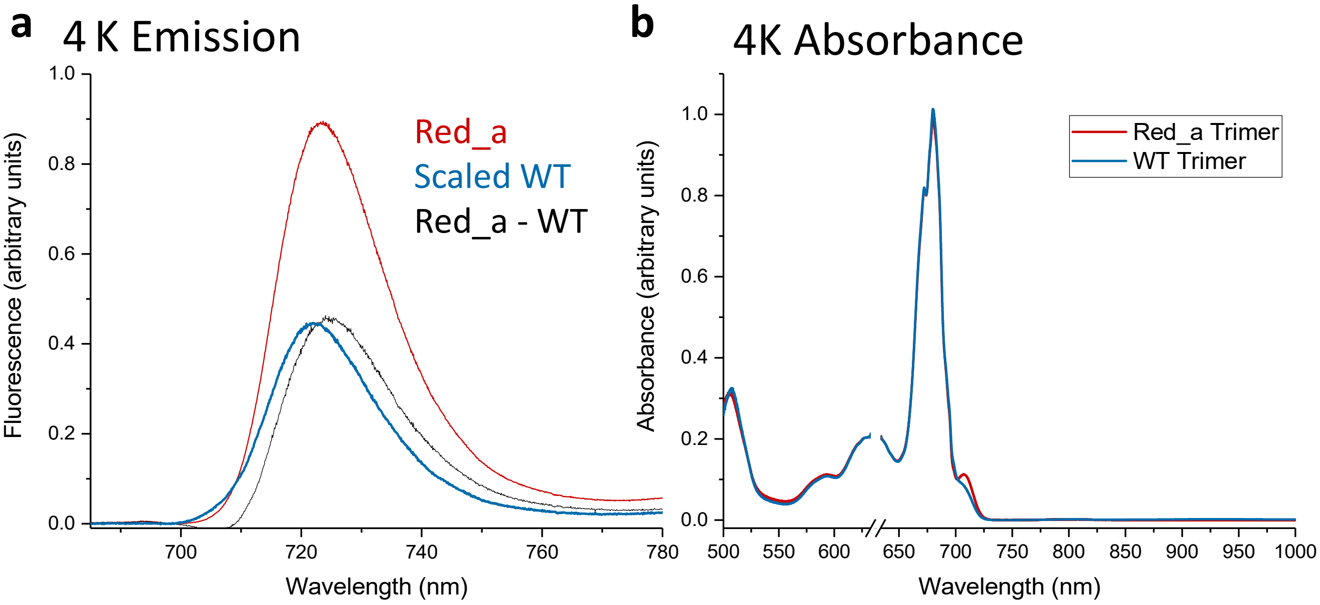

Supplementary figure 2. Low temperature absorbance and emission from WT and Red_a trimers. a. 4 K emission of WT in blue and Red_a in black together with their difference in red. **b.** 4 K absorption of wild type and Red_a PSI trimers. A signal spike centered at 632.8 nm arising from sample scattering (width of 1nm) was removed from the data.

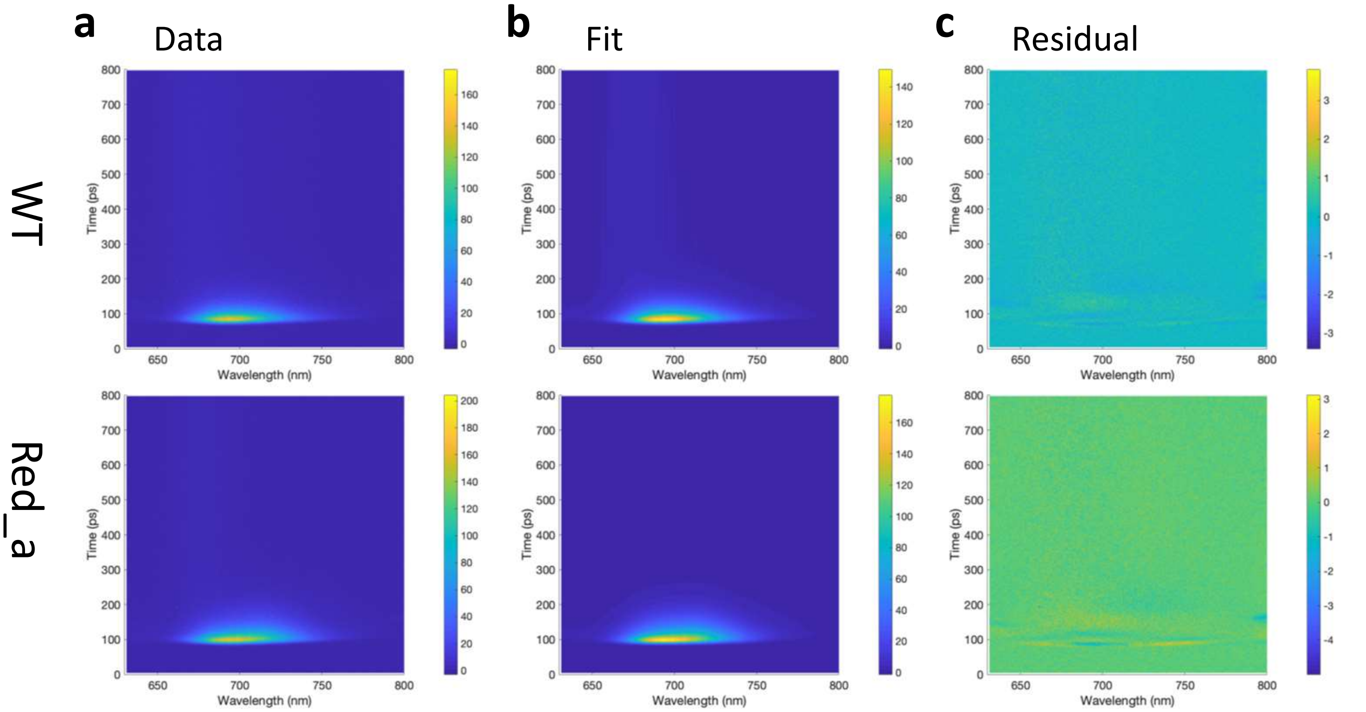

Supplementary figure 3. Time resolved Fluorescence measurements.

a. 2-D streak image of the wild type (top) and the Red_a (buttom) PSI trimers upon 400 nm excitation from 0 to 800 ps. A small number of hot pixels at the right top quadrant were corrected using mean and SD values from neighboring pixels. Individual intensity scales for each image are indicated on the right. Fit (**b**) and residuals (**c**) obtained using three decay component using global analysis.

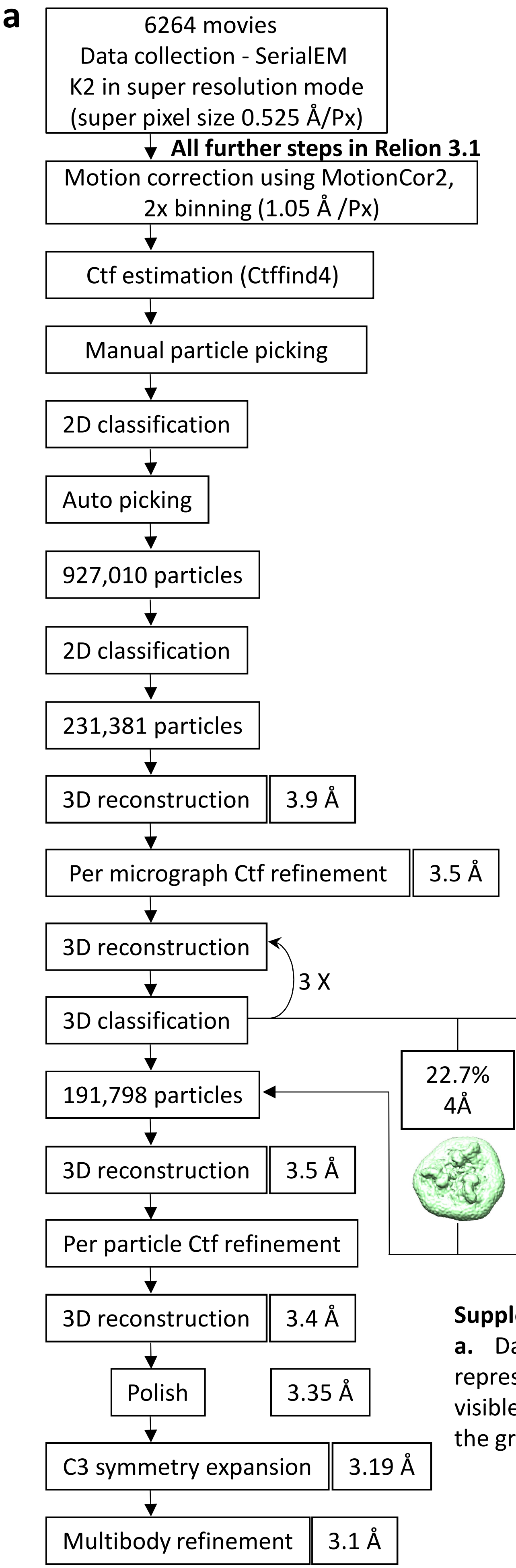

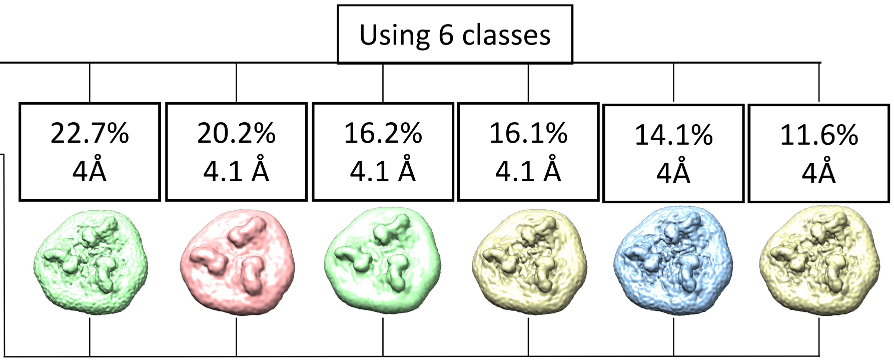

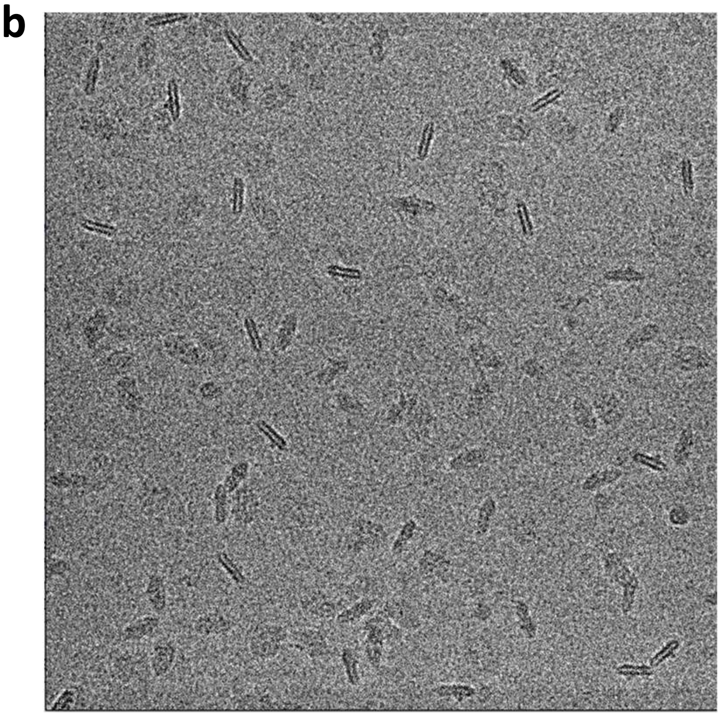

Supplementary figure 4. Image quality and processing strategy. **a.** Data collection and image processing strategy. **b**. A representative micrograph from data collection. PSI particles are visible in different orientations as projections in vitreous ice on the grid.

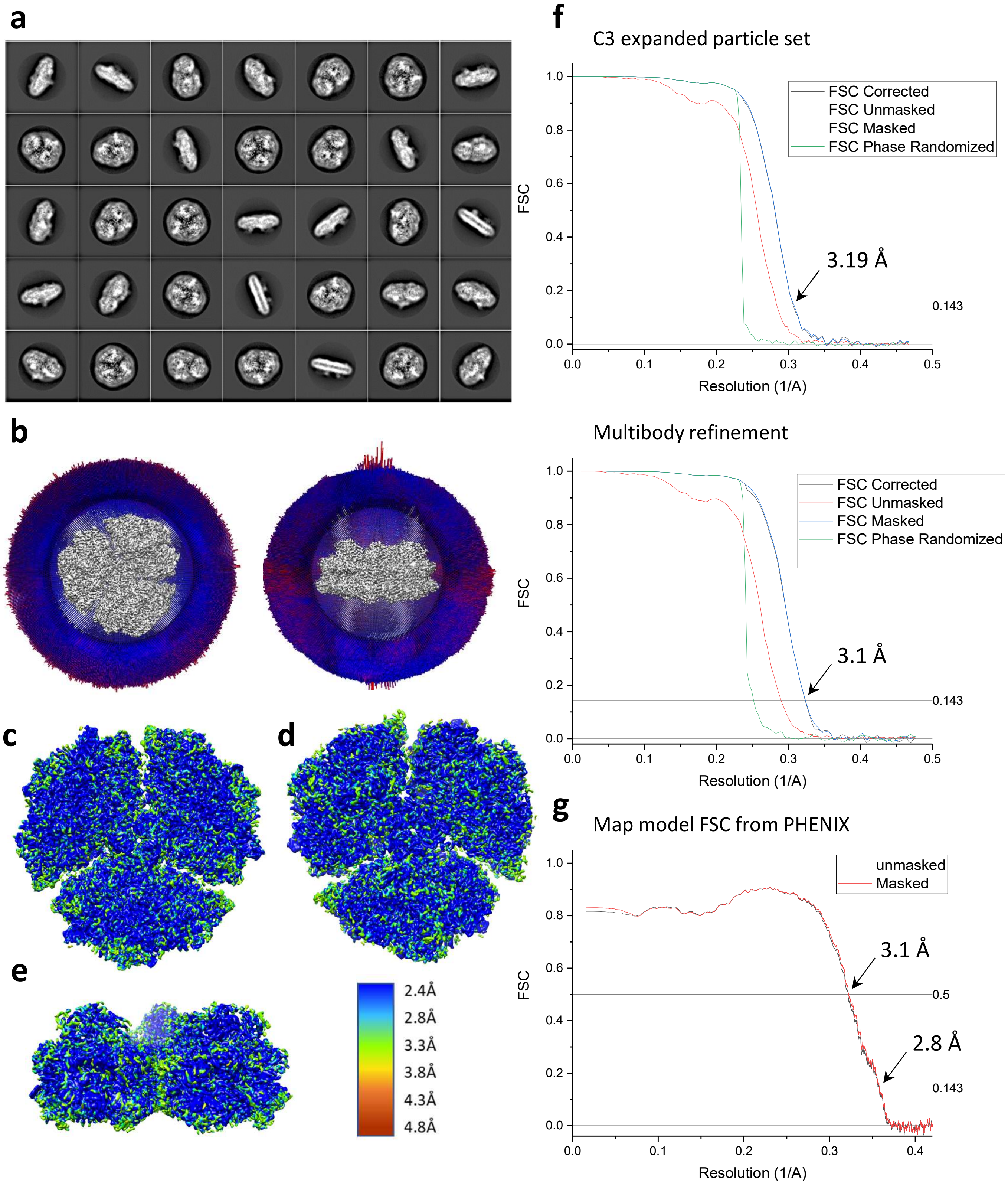

Supplementary figure 5. Resolution estimation.

a. Representative 2D class averages generated from unsupervised 2D classification. **b**. Top and side views, respectively, of the Euler angle distributions of particles obtained in the final refinement with C3 expansion. A small number of the views were removed to allow visualization of the final map. **c-e**. The final 3D map from C3 expansion process colored according to the local resolution estimates obtained from ResMap, seen from the stroma '**c**', lumen '**d**', and the membrane plane '**e**'. **f.** Plots of Fourier shell correlation (FSC) against resolution of the C3 expended particle set and of the multibody refinement. **d**. **g**. Fourier shell correlation (FSC) plot against resolution of the map to model generated by PHENIX.

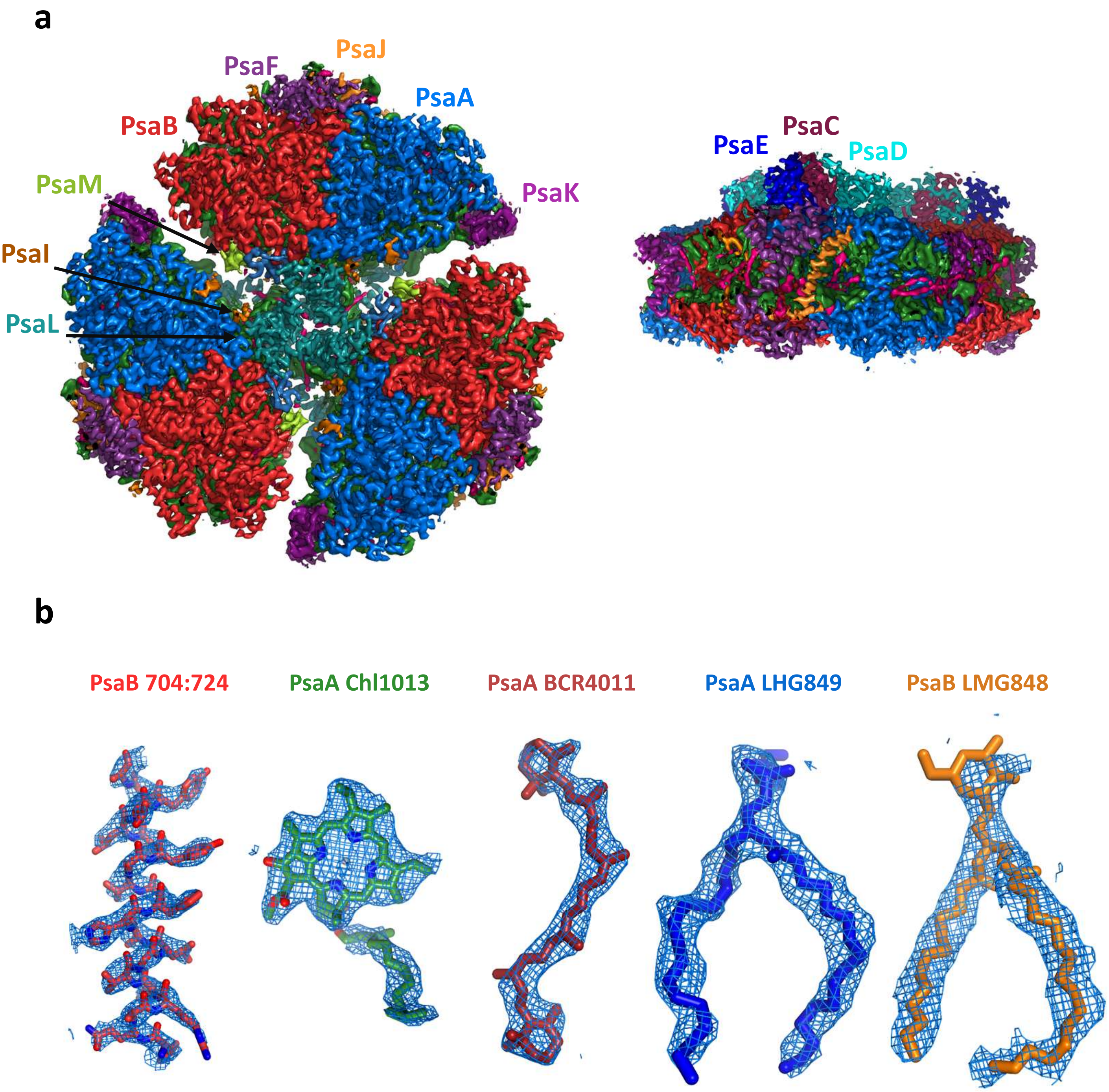

Supplementary figure 6. Final map and map examples.

a. The final 3D map colored according to individual subunits, seen from the Lumen and from the membrane plane. **b**. Representative map sections showing transmembrane helix, carotenoid, chlorophylls and lipids from PSI. The identity of each one is indicated above.

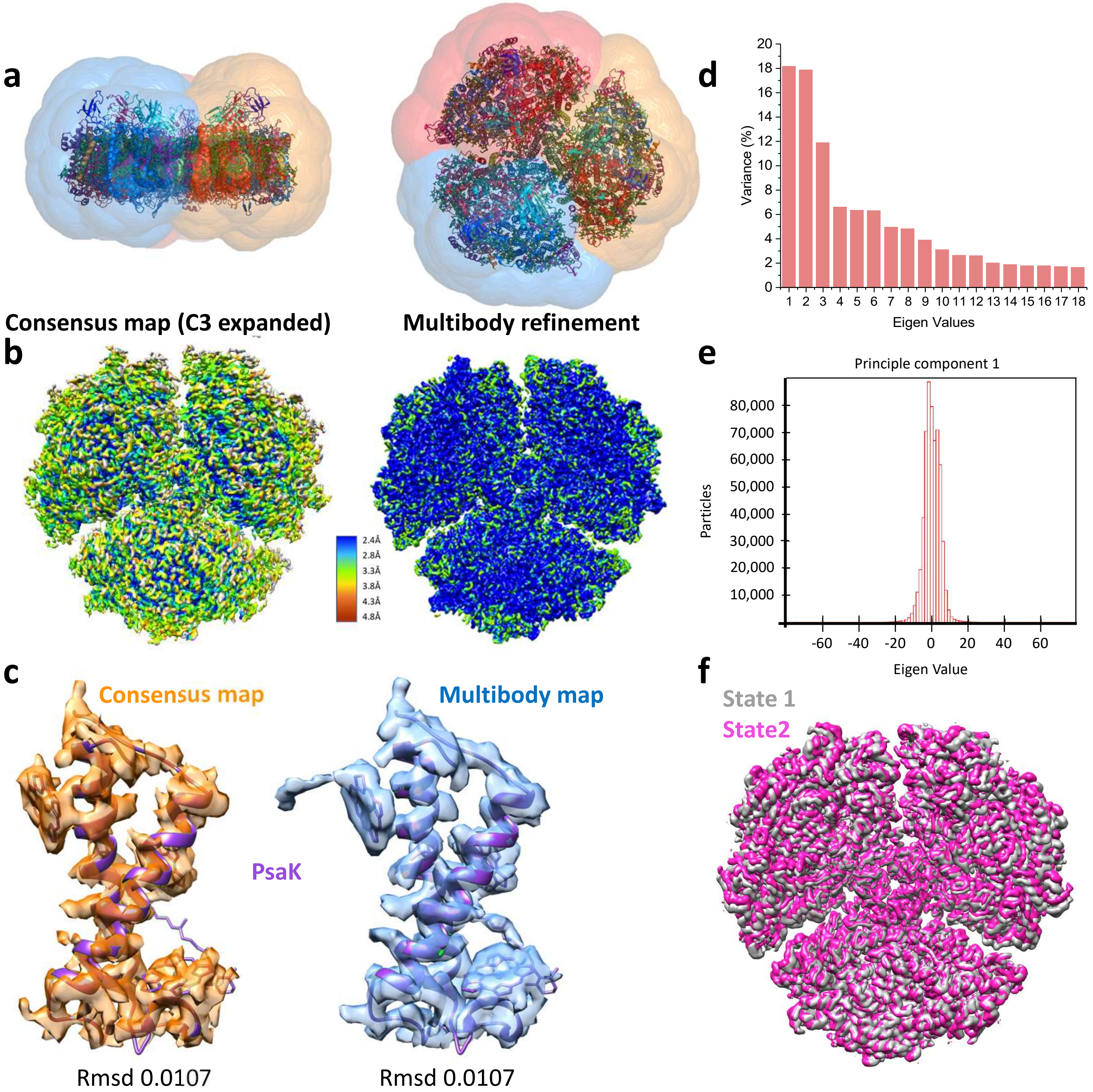

Supplementary figure 7. Multibody refinement. a. The PSI model with three transparent masks assigned for each monomer of the PSI trimer seen from the membrane plane and the lumen side. **b**. Consensus and multibody maps colored according to the local resolution estimates obtained from ResMap, seen from the lumen. **c**. Consensus and multibody maps in the PsaK area showing improvement in the map after multibody refinement. Rmsd map levels are indicated below. **d**. The values of eigenvectors that contribute to the variance in movement. **e**. The distribution of particles along the first principle component. **f.** Superposition of the two extremes states (state1 and 2) maps obtained from the first principle component in the multibody refinement procedure.

Supplementary table 1. Individual chains and ligands resolvability according to Q-scores. Scores were calculated using the MapQ plugin in uscfChimera[23].

Chain	Q score	Estimated Res (Å)
**A**	0.69	2.42
**B**	0.69	2.4
**C**	0.68	2.47
**D**	0.63	2.76
**E**	0.61	2.86
**F**	0.62	2.81
**I**	0.68	2.49
**J**	0.63	2.77
**K**	0.54	3.24
**L**	0.66	2.56
**M**	0.65	2.64
**Chlorophyll A**	0.71	
**Beta carotene**	0.69	

Supplementary table 2. Mg-mg distances and orientations at PSI interfaces.

Interface	Chain	Residue	Chain	Residue	State 1		State 2		Red_a	
					**Orientation factor [a]**	**Distance (Å)**	**Orientation factor [a]**	**Distance (Å)**	**Orientation factor [a]**	**Distance (Å)**
**BC**	B(4)	1211	1	1134	2.2	23.9	2.19	18.9	2.18	21.1
**BC**	B(5)	1212	8	1401	1.05	21.8	1.04	16.2	2.01	18.6
**BC**	B(2)	1208	1	1121	1.57	22.1	1.51	17.4	1.55	19.2
**BC**	B(3)	1209	1	1120	0.07	27.8	0.08	22.4	0.11	24.6
**BC**	B(2)	1208	1	1801	0.76	19.2	0.73	15.3	0.73	16.8
**AB**	2(12)	1211	a	1134	2.18	17.6	2.19	22	0.9	21.3
**AB**	2(13)	1212	k	1401	1.03	18.1	1.06	19.5	2	18.7
**AB**	2(10)	1208	a	1121	1.51	19.1	1.55	20.2	1.56	19.4
**AB**	2(11)	1209	a	1120	0.09	24.2	0.07	25.6	0.11	24.8
**AB**	2(10)	1208	a	1801	0.67	16.8	0.81	17.6	0.74	16.9
**CA**	b(8)	1211	A	1134	2.17	19.7	2.15	23	2.18	21.3
**CA**	b(9)	1212	K	1401	1.06	17.1	1.05	20.9	0.89	18.9
**CA**	b(6)	1208	A	1121	1.59	18	1.61	21.1	1.54	19.4
**CA**	b(7)	1209	A	1120	0.07	23	0.09	26.7	0.11	24.7
**CA**	b(6)	1208	A	1801	0.8	15.8	0.71	18.3	0.74	16.9

Supplementary table 3. Primers for the construction of Red_a muant

**Basic plasmid construction for mutations in PsaA and PsaB genes**	
PsaAB_fwd	ttgacagcttatcatcgataatgacaattagtccacccgaaag
PsaAB_rev	cccccccccccgatgggttcccggtcaac
kan_fwd	gaacccatcgggggggggggggaaagccac
kan_rev	gtggctccaatgcaggggggggggggcg
PsaBdown_fwd	cccccctgcattggagccacatctgccag
PsaBdown_rev	tagctgaacaggagggacagcactactctctctggtggc
PA15ori_fwd	ctgtccctcctgttcagc
PA15ori_rev	tatcgatgataagctgtcaaacatg
**Red_a construction**	
Red_a_fragment1_fwd	atcctgacagcattgcttccactgcttggccgaactatggcaacgtctggttacccggctggttggatgctatcaacagc
Red_a_fragment1_rev	Taattgtcattatcgatgataagctgtcaaacatg
Red_a_fragment2_fwd	Atcatcgataatgacaattagtccacccg
Red_a_fragment2_rev	gcatccaaccagccgggtaaccagacgttgccatagttcggccaagcagtggaagcaatgctgtcaggattggagagcaa

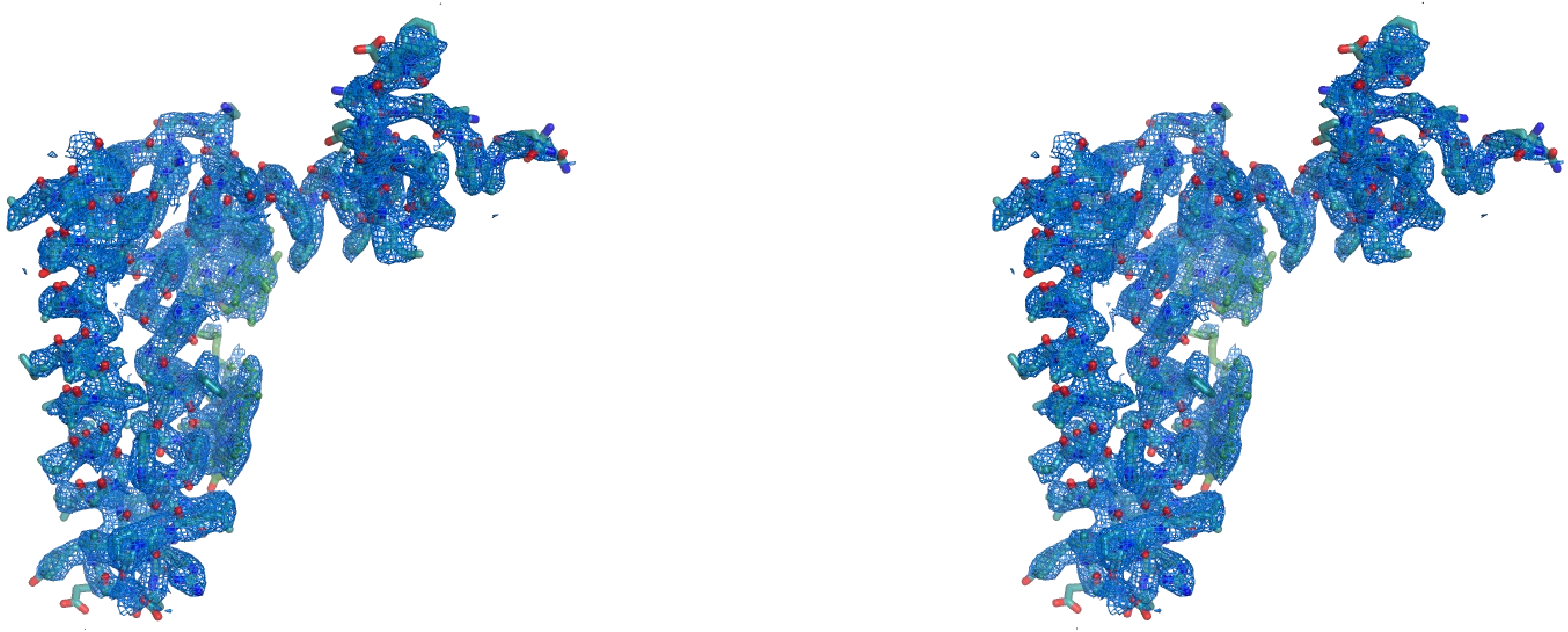

A stereo image of the cryo-EM map surrounding the PsaL subunit, contoured at 3 σ.
