# Supplementary material for: The structure of a red-shifted photosystem I reveals a red site in the core antenna
Source: Nat Commun. 2020 Oct 19;11:5279. doi: 10.1038/s41467-020-18884-w (PMC7573975; doi:10.1038/s41467-020-18884-w)
Supplement: Supplementary file 3 — Descriptions of Additional Supplementary Files [file 41467_2020_18884_MOESM3_ESM.pdf]

Descriptions of Additional Supplementary Files

Supplementary Movie 1

Description: First PCA component view from the lumen.

Supplementary Movie 2

Description: First PCA component view from the membrane side.

Supplementary Movie 3

Description: First PCA component view from the stroma.

Supplementary Movie 4

Description: Second PCA component view from the lumen.

Supplementary Movie 5

Description: Second PCA component view from the membrane side.

Supplementary Movie 6

Description: Second PCA component view from the stroma.
